# Spatial Clusters of Cancer Mortality in Brazil: A Machine Learning Modeling Approach

**DOI:** 10.3389/ijph.2023.1604789

**Published:** 2023-07-20

**Authors:** Bruno Casaes Teixeira, Tatiana Natasha Toporcov, Francisco Chiaravalloti-Neto, Alexandre Dias Porto Chiavegatto Filho

**Affiliations:** Department of Epidemiology, Faculty of Public Health, University of São Paulo, São Paulo, Brazil

**Keywords:** Brazil, cancer, machine-learning, spatial-clusters, socioeconomic

## Abstract

**Objectives:** Our aim was to test if machine learning algorithms can predict cancer mortality (CM) at an ecological level and use these results to identify statistically significant spatial clusters of excess cancer mortality (eCM).

**Methods:** Age-standardized CM was extracted from the official databases of Brazil. Predictive features included sociodemographic and health coverage variables. Machine learning algorithms were selected and trained with 70% of the data, and the performance was tested with the remaining 30%. Clusters of eCM were identified using SatScan. Additionally, separate analyses were performed for the 10 most frequent cancer types.

**Results:** The gradient boosting trees algorithm presented the highest coefficient of determination (*R* ^2^ = 0.66). For total cancer, all algorithms overlapped in the region of Bagé (27% eCM). For esophageal cancer, all algorithms overlapped in west Rio Grande do Sul (48%–96% eCM). The most significant cluster for stomach cancer was in Macapá (82% eCM). The most important variables were the percentage of the white population and residents with computers.

**Conclusion:** We found consistent and well-defined geographic regions in Brazil with significantly higher than expected cancer mortality.

## Introduction

Cancer occurrence significantly varies among different geographical locations and types of cancer. A comprehensive analysis of age-adjusted incidence rates on a global scale found that, in 2018, the incidence was 419 per 100,000 inhabitants in Oceania, 350 in North America, 217 in Latin America and the Caribbean, and 130 in Africa, as per the report by [1].

In Brazil, cancer is ranked as the second leading cause of death, resulting in 227,920 deaths in 2018, as estimated by the World Health Organization [2]. It also reports that the age-adjusted death rate due to cancer is 111 per 100,000 for men and 95 per 100,000 for women. Within Brazil, these rates fluctuate considerably, with higher rates observed in the country’s more developed southern and southeastern regions [3].

Brazil, with its vast territory and stark socioeconomic disparities, is home to a multitude of ethnic groups. Yet, its healthcare system is relatively uniform nationwide, which makes it a potentially promising setting for eco-epidemiologic modeling. Machine learning models have been utilized in diverse healthcare fields, primarily for creating individualized prediction algorithms. These include predicting the mortality risk during chemotherapy [4, 5], predicting in-hospital mortality [6, 7], and making prognostic predictions [8–10]. Yet, their application in ecological epidemiology modeling is still growing [11–13].

Spatial epidemiology methods can effectively identify disease incidence variations in relation to demographic, environmental, behavioral, socioeconomic, and genetic risk factors [14]. The scan statistic method by Kulldorff [15] for spatial clustering analysis is commonly used to identify areas with high health-related outcome rates. This technique has been employed in oncology research for diseases like lung cancer in China [16], colorectal cancer in Florida [17], and breast cancer in the United States [18].

The potential of merging scan statistics with machine learning models is an expanding area of research [19], with exciting applications in obesity [20], HIV, and mobile data [21]. It can also be pivotal in identifying clusters with unexpectedly high incidence rates based on local characteristics.

The primary objective of this study is to analyze the predictive power of sociodemographic variables for cancer mortality rates across the municipalities in Brazil. Following this, the research then intends to leverage these findings to identify clusters with unusually high cancer mortality rates, in other words, mortality rates not explained solely by local socioeconomic factors.

## Methods

### Data Collection

**FIGURE 1:**
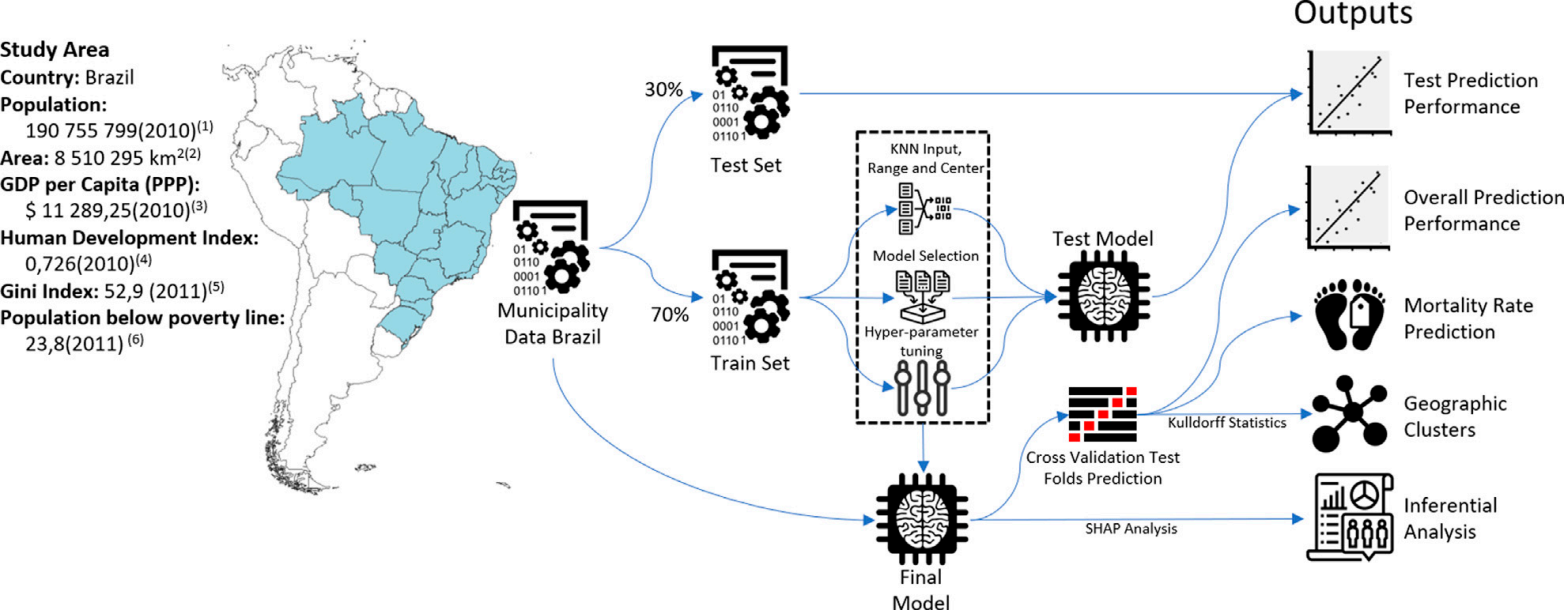
Study area and schematic diagram of methods (Spatial clusters of cancer mortality in Brazil: a machine learning modeling approach, Brazil, 2008–2016). 1) 2010 Census [24], 2) Brazilian Territory 2020 [30], 3) Gross Domestic Product (GDP) by Purchasing Power Parity (PPP) and 2017 International Dollars [47], 4) Human Development Index (HDI) [48], 5) Gini Index (World Bank Estimate) [49], and 6) Poverty headcount ratio at $5.50 a day (2011 PPP) (% of population) [50].

The schematic representation of the study is shown in Figure 1. We first extracted crude mortality data from each of the 5,565 municipalities of Brazil using the Mortality Information System (SIM) of the Ministry of Health, which has been shown to have high coverage, capturing over 95% of deaths in the Brazilian territory [22]. Cancer mortality was selected using the International Statistical Classification of Diseases and Related Health Problems (ICD)-10 codification for malignant tumors (Chapter 2 of the ICD-10) and aggregated to the Municipality level from 2007 to 2016. Age-adjusted mortality rates for each municipality were calculated using the World Health Organization Standard population from 2000 to 2025 [23].

A total of 40 sociodemographic variables focusing on income, assets, demography, and urbanization were collected from the last census (from 2010), with municipalities as the aggregated level [24]. The percentage of private healthcare coverage was obtained from the Ministry of Health [25]. Details on all variables are listed in Supplementary Annex S1. Geographical coordinates of municipalities for the spatial analysis were obtained from the Brazilian Institute of Geography and Statistics (IBGE) [24].

### Machine Learning Models

Municipalities were randomly split into train and test sets (70% and 30% of the total sample, respectively). Missing data for covariates were imputed using the K-Nearest Neighbors method, and continuous variables were standardized to the range between 0–1 using the caret package [26] from R software [27]. The train set was used to tune the hyperparameters with three repeated 10-fold cross-validation. We first tested the predictive performance of nine popular machine learning algorithms: linear regression, LASSO regression (LASSO), ridge regression, random forests (RF), extreme gradient boosting (XGB), linear support vector machines (LSVM), polynomial support vector machines (PSVM), conditional inference model tree, and decision trees. Model performance in the train set was measured with *R* ^2^ with 95% confidence intervals (95% CI), and the four best algorithms according to this metric were selected (XGB, RF, pSVM, and LASSO).

We performed hyperparameter selection for each algorithm with a 10-fold cross-validation algorithm trained with a random search algorithm using three repetitions with standard variations provided by the caret package [26]. Model performance was measured solely in the test set (Supplementary Annex S3).

After selecting the best-performing combination of hyperparameters of the algorithms, each was trained on the whole set with 10-fold cross-validation, and the results of the test folds were the predictive values for the next steps of the analysis to guarantee that every municipality has a test set result. After selecting the best-performing model, a variable importance analysis was performed with SHAP (Shapley Additive Explanation) [28].

### Geographical Analysis

To identify geographical clusters of higher-than-expected cancer mortality rates (not explained by sociodemographic characteristics), the prediction of the machine learning algorithms was used in the Kulldorff scan statistic [15] as the expected incidence rate value in association with the actual incidence rate. Additionally, the analysis incorporated the projected population [29] and municipalities centroids obtained from IBGE [30].

The Kulldorf scan statistic works by moving a varying-size circular window across a study region and, for each location, comparing the observed and expected number of cases within and outside the window using a likelihood ratio test. Under the null hypothesis, the disease risk is the same within and outside the window, while the alternative hypothesis assumes a higher risk within. The window that maximizes the likelihood ratio is considered the most likely cluster. The statistical significance of the detected clusters is then assessed using Monte Carlo simulation [15].

The only parameter in the Kulldorff scan statistic is the maximum cluster size, which is determined by spatial territory or by the population at risk. Kulldorff and Nagarwalla [31] reported that a scan window no higher than 50% of the at-risk population is the ideal rule of thumb to avoid negative cluster detection. Due to the unusually low and highly variable population density in Brazil, a cluster size of 0.3% of the total Brazilian population was considered to capture a meaningful territorial extension. It is important to note that, for a country like Brazil with sparsely populated rural areas, clusters with larger populations could potentially distort the analysis by generating clusters disproportionately extensive.

### Sub Analysis of Specific Types of Cancer

The analysis was first performed for all cancers combined (Chapter 2 of ICD-10), and then specific analyses were performed for the 10 types of cancer with the highest number of deaths (Supplementary Annex S2).

### Ethics

The data used in this analysis is freely available in the public domain by the Ministry of Health of Brazil, thus not requiring an ethics committee approval for access, analysis, and publication.

## Results

In the algorithm selection phase, best performing algorithms were RF (*R* ^2^ = 0.651, 95% CI = 0.640–0.662, and Root Mean Squared Error–RMSE = 15.4), XGB (*R* ^2^ = 0.626, 95% CI = 0.615–0.637, and RMSE = 15.8), pSVM (*R* ^2^ = 0.599, 95% CI = 0.576–0.622, and RMSE = 16.5), and LASSO (*R* ^2^ = 0.588, 95% CI = 0.578–0.598, and RMSE = 16.6). The results for every algorithm in the selection phase are presented in Supplementary Annex S3.

After hyperparameter tuning, the XGB model presented the best performance for predicting total cancer mortality rates (*R* ^2^ = 0.65 in the test set and 0.66 in the whole database; RMSE = 15.2 and 15.1, respectively). A correlation plot for the XGB model is shown in Figure 3A.

**FIGURE 2:**
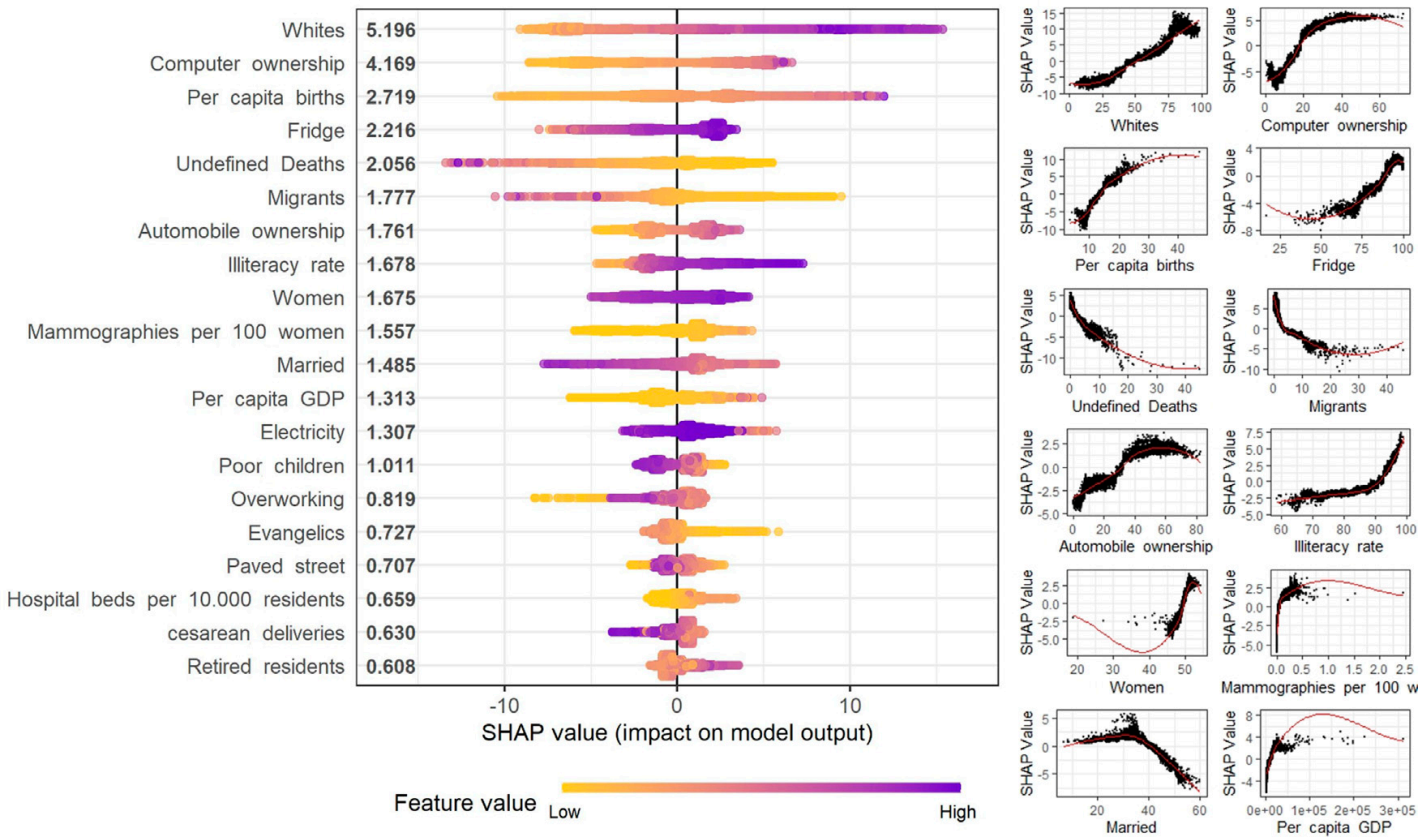
SHapley Additive exPlanations (SHAP) analysis of the top 12 contributing variables contribution to the extreme Gradient Boosting model for Cancer Mortality rate using Sociodemographic (Spatial clusters of cancer mortality in Brazil: a machine learning modeling approach, Brazil, 2008–2016).

The SHAP analysis of the XGB model shows that the most important predictive variable was the percentage of white residents (Figure 2). This variable had a positive relationship with the prediction in the entire distribution range. The second most important variable was computer ownership. This variable had a non-linear relationship with a growing contribution until around 30%, then stabilizing above this value. Per capita births were the third most important variable, with a positive relationship until up to 25 births per thousand inhabitants, also stabilizing above this rate.

**FIGURE 3:**
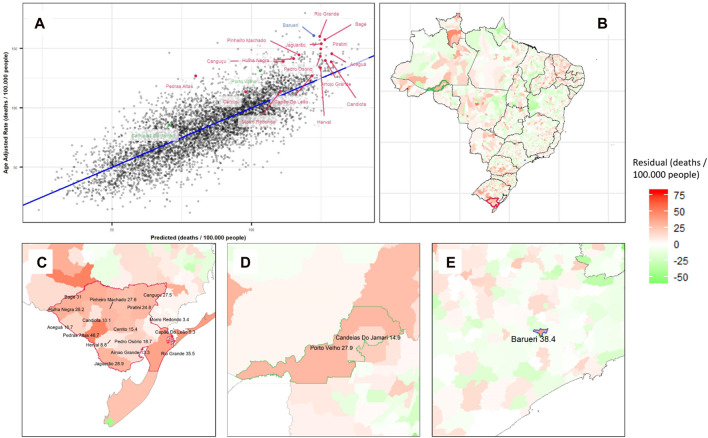
Extreme gradient boosting model results for predicting cancer mortality rates and its residuals, showing the portion of mortality not explained by local characteristics: **(A)** Correlation plot with *R* ^2^ 0.66 for Cancer Adjusted Mortality per 100,000 people in Brazilian Municipalities (colored by Kulldorff clusters). **(B)** Residuals plotted on a Brazilian map with clusters identified by color. Zoom in Barueri **(C)**, Bagé-Rio Grande Cluster **(D)**, and Porto Velho and Surroundings Cluster **(E)** (Spatial clusters of cancer mortality in Brazil: a machine learning modeling approach, Brazil, 2008–2016).

A total of three geographic clusters of residual mortality rates (high predictive error in the overall set) were identified by the Kulldorff Statistics (Figure 3). The primary cluster, with the lowest *p*-value (*p* = 0.001), was the region between Rio Grande and Bagé in the State of Rio Grande do Sul (RS) with an excess of 28.6 deaths per 100,000 residents (*p* = 0.001). The secondary cluster was the region of Porto Velho in the State of Rondônia (RO) with an excess of 27.3 deaths per 100,000 people (*p* = 0.001), and the third was the city of Barueri in the State of São Paulo (SP) with an excess mortality rate of 38.4 deaths per 100,000 people (*p* = 0.001) (Table 1).

**TABLE 1:** Cancer mortality rates by Kulldorff statistic cluster and municipality by the extreme gradient boosting algorithm (Spatial clusters of cancer mortality in Brazil: a machine learning modeling approach, Brazil, 2008–2016).

Cluster/Municipality	Adjusted rate (deaths per 100,000)	Predicted rate (deaths per 100,000)	Residual (deaths per 100,000)	Population (thousands)	Additional cases
Cluster 1	**151.3**	**122.7**	**28.6**	**538.2**	**154.1**
Rio Grande	160.0	124.5	35.5	205.2	72.8
Bagé	157.3	126.3	31.0	121.0	37.5
Canguçu	138.9	111.4	27.5	55.3	15.2
Jaguarão	154.0	125.1	28.9	28.6	8.3
Capão Do Leão	127.0	121.7	5.3	25.2	1.3
Piratini	149.6	124.9	24.8	20.6	5.1
Arroio Grande	139.8	126.5	13.3	19.0	2.5
Pinheiro Machado	144.6	117.0	27.6	13.1	3.6
Candiota	138.6	128.6	10.1	9.2	0.9
Pedro Osório	143.4	124.7	18.7	8.0	1.5
Herval	133.6	124.8	8.8	7.0	0.6
Cerrito	113.3	97.9	15.4	6.5	1.0
Morro Redondo	113.8	110.4	3.4	6.5	0.2
Hulha Negra	141.3	115.1	26.2	6.3	1.7
Aceguá	145.5	128.8	16.7	4.6	0.8
Pedras Altas	126.8	80.1	46.7	2.2	1.0
Cluster 2	**129.0**	**101.7**	**27.3**	**498.0**	**136.0**
Porto Velho	131.0	103.2	27.9	475.7	132.7
Candeias Do Jamari	85.4	70.5	14.9	22.4	3.3
Cluster 3	**160.7**	**122.4**	**38.4**	**253.9**	**97.4**
Barueri	160.7	122.4	38.4	253.9	97.4
Brazil—Others	**98.5**	**98.2**	**0.3**	**197,908.7**	**584.6**

Bold represents the totals for each cluster.

When comparing different prediction algorithms for each specific cancer type, the RF and XGB algorithms had the highest overall predictive performance. Using the Kulldorff Statistic, the LASSO model identified the highest number of clusters. No significant clusters were identified for liver, pancreatic, breast, prostate, and brain cancers for any of the models (Supplementary Annex S4).

**FIGURE 4:**
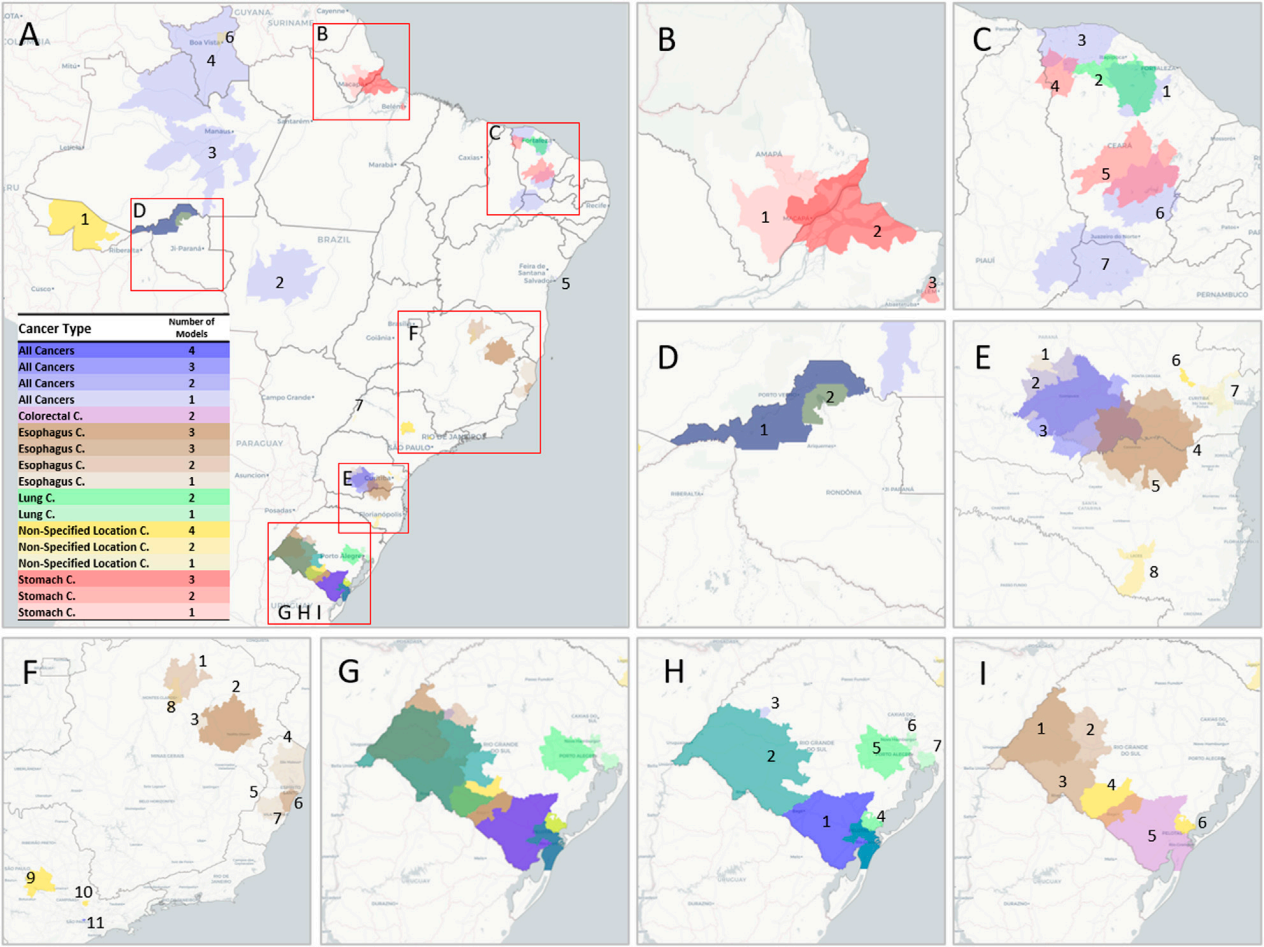
Geographic cluster location for various cancer types and regions. **(A)** Brazil. **(B)** Macapá region with two overlapping clusters of Stomach Cancer. **(C)** Ceará State with different clusters for Total Cancer, Lung Cancer, and Stomach Cancer. **(D)** Porto Velho Region with four overlapping clusters for Total Cancer, Lung Cancer, and Stomach Cancer. **(E)** Regions in the State of Paraná with different combinations of clusters for Total Cancer, Esophagus Cancer, and Non-Specified Location Cancer. **(F)** Southeast Brazil with seven clusters for Esophagus Cancer. **(G)** Rio Grande do Sul State with a varied combination of clusters for different types, specified for Total and Lung Cancer **(H)** and Esophagus, Colorectal, and Non-Specified Location Cancers **(I)** (interactive version available at https://labdaps.github.io/cancerclusters.html **)** (Spatial clusters of cancer mortality in Brazil: a machine learning modeling approach, Brazil, 2008–2016).

Figure 4 and Table 2 detail the overlapping clusters identified by each model and cancer type. For total cancer, a cluster composed of 16 cities (Figure 4H1) was identified by four algorithms in the region between Bagé and Rio Grande, and in this same region, a cluster for colorectal cancer was identified by two models (Figure 4I5). Several overlapping clusters of total, lung, and stomach cancer were identified in the Region of Porto Velho and surrounding areas (Figure 4D). In the region of Macapá, two clusters for stomach cancer were identified: the first, identified by three models (Figure 4B2), intersected the city of Macapá, and the second, around Santana, was identified by one model (Figure 4B1). Multiple clusters were identified in the State of Ceará for different cancer types by the LASSO and pSVM models (Figure 4C).

**TABLE 2:** Geographic cluster details for various cancer types and regions (Spatial clusters of cancer mortality in Brazil: a machine learning modeling approach, Brazil, 2008–2016).

Cancer type	Number of models	Figure 4 location	Models	Number of cities	Population	Mean estimated	Number of cases	Cluster *p*-value range	Mean excess cases (%)
Total Cancer	4	H1	XGB, RF, SVM, LASSO	16	538.220	643	815	0.001–0.002	27
Total Cancer	3	F11	XGB, RF, LASSO	1	253.877	309	408	0.001–0.005	32
Total Cancer	2	D1	SVM, LASSO	1	475.691	447	623	0.001–0.001	40
Total Cancer	2	E3	SVM, LASSO	20	587.156	641	820	0.001–0.001	28
Total Cancer	1	C1	LASSO	18	500.042	334	445	0.001–0.001	33
Total Cancer	1	A4	LASSO	18	556.312	416	550	0.001–0.001	32
Total Cancer	1	H2	LASSO	16	574.415	635	806	0.001–0.001	27
Total Cancer	1	C3	LASSO	23	592.999	392	496	0.002–0.002	27
Total Cancer	1	C7	LASSO	32	539.167	356	442	0.046–0.046	24
Total Cancer	1	C6	LASSO	26	586.597	428	537	0.002–0.002	25
Total Cancer	1	A2	LASSO	22	474.579	425	544	0.001–0.001	28
Total Cancer	1	A5	LASSO	2	308.748	296	375	0.047–0.047	27
Total Cancer	1	D2	XGB	2	498.045	507	642	0.001–0.001	27
Total Cancer	1	A3	RF	12	446.095	236	315	0.04–0.04	33
Total Cancer	1	E2	RF	13	401.607	455	564	0.047–0.047	24
Total Cancer	1	H (2, 3)	SVM	17	576.921	641	809	0.003–0.003	26
Colorectal C.	2	I5	SVM, LASSO	16	538.220	47	79	0.001–0.001	70
Esophagus C.	3	F2	XGB, RF, LASSO	28	571.144	41	61	0.002–0.007	50
Esophagus C.	3	E4	XGB, RF, LASSO	28	565.803	45	80	0.001–0.001	76
Esophagus C.	3	F6	XGB, RF, LASSO	5	586.686	26	47	0.001–0.003	78
Esophagus C.	2	F1	RF, SVM	10	573.875	31	47	0.033–0.035	52
Esophagus C.	2	I (1, 2)	XGB, RF	16	584.172	44	65	0.001–0.004	48
Esophagus C.	1	E5	SVM	27	583.542	44	80	0.001–0.001	82
Esophagus C.	1	E1	SVM	15	431.730	32	48	0.033–0.033	50
Esophagus C.	1	F	XGB	2	443.871	21	37	0.013–0.013	76
Esophagus C.	1	F3	SVM	31	591.580	47	64	0.017–0.017	36
Esophagus C.	1	F4	LASSO	17	587.888	27	45	0.031–0.031	67
Esophagus C.	1	I (1, 3)	LASSO	10	476.257	28	55	0.001–0.001	96
Esophagus C.	1	F (5, 7)	SVM	9	551.007	29	46	0.015–0.015	59
Esophagus C.	1	F7	SVM	2	522.051	14	31	0.002–0.002	121
Esophagus C.	1	I1	SVM	6	323.479	21	39	0.004–0.004	86
Esophagus C.	1	F7	SVM	1	353.043	8	21	0.013–0.013	163
Lung C.	2	H2	SVM, LASSO	16	574.415	97	152	0.001–0.001	57
Lung C.	2	H4	SVM, LASSO	6	591.722	107	161	0.001–0.001	50
Lung C.	2	H5	SVM, LASSO	20	506.420	100	146	0.002–0.007	46
Lung C.	2	D2	SVM, LASSO	2	498.045	59	99	0.002–0.002	68
Lung C.	2	C2	SVM, LASSO	16	555.057	40	70	0.015–0.019	75
Lung C.	1	H6	LASSO	9	524.379	106	147	0.044–0.044	39
Lung C.	1	H7	LASSO	2	452.728	86	124	0.047–0.047	44
Non-Specified Location C.	4	F9	XGB, RF, SVM, LASSO	16	422.324	24	37	0.013–0.037	56
Non-Specified Location C.	4	A1	XGB, RF, SVM, LASSO	9	508.427	19	37	0.001–0.001	100
Non-Specified Location C.	4	I6	XGB, RF, SVM, LASSO	1	340.257	16	35	0.001–0.001	122
Non-Specified Location C.	4	I4	XGB, RF, SVM, LASSO	3	168.828	7	26	0.001–0.001	285
Non-Specified Location C.	4	E6	XGB, RF, SVM, LASSO	1	25.389	1	14	0.001–0.001	1,300
Non-Specified Location C.	4	F10	XGB, RF, SVM, LASSO	1	108.145	4	15	0.001–0.003	275
Non-Specified Location C.	2	A6	SVM, LASSO	1	303.002	7	19	0.001–0.029	171
Non-Specified Location C.	2	E8	RF, SVM	1	159.076	7	17	0.023–0.04	162
Non-Specified Location C.	1	F8	RF	1	381.407	15	27	0.019–0.019	80
Non-Specified Location C.	1	A7	SVM	1	39.190	1	8	0.01–0.01	700
Non-Specified Location C.	1	E7	SVM	6	524.942	20	35	0.013–0.013	75
Stomach C.	3	B2	XGB, SVM, LASSO	5	597.394	51	92	0.001–0.022	82
Stomach C.	2	C4	SVM, LASSO	13	364.233	27	50	0.021–0.028	85
Stomach C.	2	D2	SVM, LASSO	2	498.045	33	58	0.017–0.023	76
Stomach C.	2	C5	SVM, LASSO	17	585.956	38	65	0.011–0.014	71
Stomach C.	2	B3	SVM, LASSO	2	594.948	46	80	0.001–0.002	74
Stomach C.	1	B1	RF	5	577.729	57	90	0.006–0.006	58

Regarding esophageal cancer, four models identified two overlapping clusters in the region between Parana and Santa Catarina (Figure 4: E4 and E5). Three overlapping clusters were identified in the western region of the State of Rio Grande do Sul (Figure 4: I1, I2, and I3), and a similar situation was identified in the region around the city of Teófilo Otoni (Figure 4: F2 and F3). Four clusters were identified in the State of Espírito Santo (Figure 4: F4, F5, F6, and F7).

The mean excess mortality rate not explainable by sociodemographic characteristics was highest for non-specified cancer (weighted mean: 126% ± weighted standard deviation: 143% with weights on expected cases), followed by stomach cancer (73% ± 10%), colorectal cancer (one cluster 70%), esophagus cancer (67% ± 24%), lung cancer (51% ± 11%), and total cancer (28% ± 4%).

## Discussion

Utilizing solely socioeconomic factors and healthcare coverage parameters, machine learning algorithms demonstrated a high overall success rate in predicting cancer mortality. Cancer mortality is a consequence of its incidence and lethality, and both have a strong association with socioeconomic characteristics [32, 33]. Therefore, machine learning algorithms could potentially offer a superior modeling approach compared to conventional statistical methods. We used the predicted values to identify statistically significant clusters of excess cancer mortality (i.e., higher rates in comparison to the expected rate given its sociodemographic characteristics) throughout Brazil. There were consistent and significant cluster overlaps for the different algorithms, especially in the southern and northern regions of the country.

The area between Bagé and Rio Grande (Figure 4H1), the southernmost region of Brazil, was identified by all models for both total cancer and colorectal cancer. Lung cancer clusters were also particularly common in this state, with three different clusters around the capital Porto Alegre, one cluster in Pelotas/Rio Grande, and another in the western area of the State. For stomach cancer, the region of Macapá in Amapá State, Northern Brazil, showed an 82% excess in mortality in addition to two other clusters in the state of Ceará with excess mortality of 85% in the region around Barreiros and 71% around Piquet Carneiro, both in the rural part of the state. These cancer types are etiologically related to tobacco smoking [34]. In alignment with this, Macapá, Porto Alegre, and Porto Velho, the state capitals with the highest recorded rates of smoking habits among men [35], coincide with some of these identified cancer clusters. A recent study identified several clusters for gastric cancer in Brazil, one of them in the state of Ceará [36], but it is important to note that this study did not apply an adjustment for sociodemographic characteristics, which could impact some clusters, especially in the southern regions of Brazil. A cluster analysis of stomach cancer cases in Central America identified a possible association with a germline, as well as a hotspot for *Helicobacter pylori* infection, which should be a focus of future epidemiologic research in this region [37].

Although clusters of lung cancer in Rio Grande do Sul and stomach cancer in Amapá are geographically consistent with cancer incidence analyses of the National Institute of Cancer (INCA) [3], high incidence rates do not necessarily coincide with areas with an anomalous number of cases as the large variance of sociodemographic characteristics throughout Brazil could mean that even high cancer rates are within the expected value, given these local characteristics. Our study, by first predicting the cancer mortality rate of Brazilian municipalities using sociodemographic characteristics (and showing that they have a high predictive ability), was then able to identify spatial clusters with higher-than-expected cancer mortality rates considering socioeconomic and healthcare coverage factors.

It is important to note that no significant clusters were found for breast, prostate, liver, pancreatic, and brain cancers. One possible reason is that some of these specific cancers have low incidence and are, therefore, amenable to random local variations that decrease the predictive ability of the machine learning models. Another possibility is that these cancers are less significantly affected by other factors beyond sociodemographic characteristics. Eleven clusters of non-specified location cancer were also found, six of them by all four models tested. These clusters may indicate regions lacking the specialization of mortality registration services in identifying specific tumors.

Even though etiological reasoning regarding increased cancer mortality rates is beyond the scope of this study, we strongly advocate for further research to gather new local data and validate our findings. Notably, most of the cancer types with clusters identified in this study are associated with preventable risk factors [34]. For instance, tobacco smoking, the primary risk factor for lung and esophageal cancers [34], has seen a reduction due to effective anti-tobacco campaigns [38]. It is important, however, to note that part of tobacco consumption influence in the local areas may have been attenuated due to its relation to socioeconomic factors [39].

Further areas of inquiry could involve cultural practices unique to Brazil’s southern region, such as the consumption of hot mate tea and frequent barbecuing, given their observed associations with gastric [40] and lung [34] cancers, respectively. Furthermore, the most important risk factor for gastric cancer is the presence of *H. Pylori* [34], and its screening has been shown to reduce gastric cancer incidence by 35% [41] and in a cost-effective way [42]. Using spatial clustering methods associated with machine learning modeling could potentially enhance geography-targeted screening, campaigns, and epidemiological field studies.

Variable importance analysis of the machine learning algorithms found that computer ownership, automobile ownership, electricity coverage, percentage of houses with fridges, and literacy rate increased the probability of a high prediction of cancer mortality rates. Socioeconomic factors have been associated with cancer incidence and mortality, especially given that high-income individuals are able to treat competing risk factors such as cardiovascular diseases and diabetes, as well as due to the presence of differences in dietary and lifestyle behaviors [43–45].

There are important limitations to this study. First, it was not able to provide a reason for the excessive mortality rates found. However, it can provide essential guidance for future epidemiological field research regarding environmental, genetic, behavioral, and socioeconomic risk factors [14]. Second, our study focused on mortality rather than incidence because Brazil lacks a nationwide, population-based cancer registry. Mortality data, being of higher quality and available at the municipal level, was used instead. While mortality can serve as a reasonable proxy for incidence [46], it might also encompass other sources of variability, particularly for cancer types associated with higher survival rates. Third, all the covariates are solely from point-in-time data from the last Brazilian census. However, considering that cancer is a chronic disease, it is likely that the fluctuation over a small number of years would not be very significant. Lastly, the quality of the mortality data could vary among the different cities. In order to try to mitigate this potential concern, we added a proxy for data quality in the model (the rate of unidentified cause mortality), but there could still be some remaining issues regarding data quality heterogeneity.

Combining clustering statistics with machine learning modeling offers a promising and adaptable tool applicable to a wide array of outcomes. This study identified numerous clusters of elevated mortality across various regions of Brazil for lung, stomach, esophageal, colorectal, and overall cancer. These findings not only highlight potential areas for further epidemiological field studies but also provide guidance for targeted healthcare interventions.
