# Supplementary material for: Spatial Clusters of Cancer Mortality in Brazil: A Machine Learning Modeling Approach
Source: Int J Public Health. 2023 Jul 20;68:1604789. doi: 10.3389/ijph.2023.1604789 (PMC10397398; doi:10.3389/ijph.2023.1604789)
Supplement: Supplementary file 1 [file DataSheet1.docx]

# Supplementary Materials

Annex 1 - Sociodemographic varialbles summary statistics. (Spatial clusters of cancer mortality in Brazil: a machine learning modelling approach, Brazil, 2008-2016)

|  | Overall (N=5565) | | Missing |
| --- | --- | --- | --- |
| **Variable** | **Mean (SD)** | **Range** | **N (%)** |
| Life expectance | 73.089 (2.681) | 65.300 - 78.640 | 0 |
| Residents | 34277.772 (203112.622) | 805.000 - 11253503.000 | 0 |
| Median age | 29.025 (4.367) | 13.590 - 46.740 | 0 |
| Elderly | 12.090 (3.286) | 2.550 - 29.190 | 0 |
| Dependency ratio | 60.255 (9.010) | 15.940 - 122.980 | 0 |
| Women | 49.504 (1.569) | 18.910 - 54.240 | 0 |
| Per capita births | 13.936 (3.609) | 3.400 - 47.460 | 0 |
| Married | 36.535 (8.683) | 6.550 - 64.040 | 0 |
| Evangelics | 17.100 (9.465) | 0.420 - 85.840 | 0 |
| Disability rate | 24.565 (4.746) | 2.540 - 44.330 | 0 |
| Municipal density | 108.202 (572.445) | 0.130 - 13024.600 | 0 |
| Urban area | 63.835 (22.036) | 4.180 - 100.000 | 0 |
| Fridge | 88.620 (11.627) | 16.660 - 100.000 | 0 |
| Computer ownership | 21.330 (13.982) | 0.440 - 72.700 | 0 |
| Automobile ownership | 31.869 (19.948) | 0.000 - 90.720 | 0 |
| Household density | 3.391 (0.431) | 2.560 - 6.920 | 0 |
| Favela (slums) residents | 0.132 (0.402) | 0.000 - 9.880 | 0 |
| Electricity | 97.076 (5.801) | 29.520 - 100.000 | 0 |
| Green spaces | 0.433 (0.245) | 0.000 - 0.980 | 0 |
| Paved street | 0.465 (0.236) | 0.000 - 0.990 | 0 |
| Whites | 46.948 (24.049) | 0.860 - 99.160 | 0 |
| Illiteracy rate | 85.259 (8.936) | 58.400 - 99.100 | 0 |
| College education | 5.507 (3.275) | 0.280 - 33.840 | 0 |
| Highschool completion | 16.183 (6.051) | 1.860 - 47.470 | 0 |
| Migrants | 5.501 (4.476) | 0.000 - 45.750 | 0 |
| Foreigners | 0.076 (0.546) | 0.000 - 37.720 | 0 |
| Median income | 566.155 (265.771) | 128.770 - 2210.720 | 0 |
| Unemployment | 3.752 (1.993) | 0.000 - 16.990 | 0 |
| Child labor | 13.026 (8.301) | 0.000 - 72.090 | 0 |
| Retired residents | 16.712 (4.669) | 2.320 - 40.180 | 0 |
| Overworking | 28.615 (10.956) | 0.900 - 73.090 | 0 |
| Poor children | 59.951 (22.496) | 2.440 - 95.530 | 0 |
| Per capita GDP | 12587.939 (14676.853) | 2257.990 - 312257.340 | 0 |
| Gini coefficient | 0.503 (0.066) | 0.280 - 0.810 | 0 |
| Bolsa Familia Coverage | 75.927 (18.751) | 0.000 - 100.000 | 0 |
| Communiting | 10.816 (9.815) | 0.000 - 69.330 | 0 |
| Private Insurance | 9.120 (11.848) | 9.120 (11.848) | 6 (0.11) |
| Mammographies per 100 women | 0.104 (0.112) | 0.000 - 2.460 | 0 |
| Oral Health Strategy coverage | 65.462 (37.253) | 65.462 (37.253) | 1 (0.02) |
| Primary health coverage for poor residents | 79.617 (27.193) | 0.000 - 100.000 | 0 |
| Vaccination coverage | 79.098 (10.723) | 0.000 - 182.370 | 0 |
| Low birth weight | 7.677 (3.473) | 0.000 - 40.000 | 0 |
| Hospital beds per 10.000 residents | 1.546 (1.843) | 0.000 - 24.540 | 0 |
| Family Health Strategy teams per 10.000 residents | 0.296 (0.141) | 0.000 - 1.230 | 0 |
| Ultrasound machines. per 10.000 live births | 4.573 (8.390) | 0.000 - 125.000 | 0 |
| cesarean deliveries | 50.896 (18.411) | 3.330 - 100.000 | 0 |
| Xray machines per 10.000 residents | 0.111 (0.144) | 0.000 - 1.460 | 0 |
| Life support equipment per 10.000 residents | 0.303 (0.369) | 0.000 - 3.250 | 0 |
| Undefined Deaths | 3.134 (3.284) | 0.000 - 45.238 | 0 |

Annex 2 - Top-10 Cancer Types by mortality (2007 - 2016) and ICD-10 codes. (Spatial clusters of cancer mortality in Brazil: a machine learning modelling approach, Brazil, 2008-2016)

| **Cancer Type** | **Number of Deaths accounted** |
| --- | --- |
| C34 - Lung Cancer | 212.379 |
| C18-C21 - Colorectal Cancer | 132.802 |
| C50 - Breast Cancer | 125.008 |
| C16 - Stomach Cancer | 123.188 |
| C61 - Prostate Cancer | 120.999 |
| C22 - Liver Cancer | 76.704 |
| C25 - Pancreatic Cancer | 74.123 |
| C15 - Esophagus Cancer | 70.386 |
| C71 - Brain Cancer | 64.741 |
| C80 – Non-Specified Location Cancer | 56.340 |

Annex 3 - Resample plot of model performance in the train set with 95% Confidence Intervals. (Spatial clusters of cancer mortality in Brazil: a machine learning modelling approach, Brazil, 2008-2016)


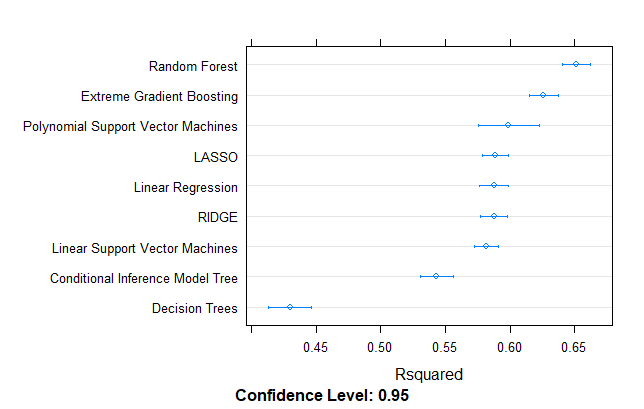


Annex 4 - Prediction Accuracy and Clusters Identified by the Kulldorff Statistic for different Machine Learning algorithms and Cancer types (Spatial clusters of cancer mortality in Brazil: a machine learning modelling approach, Brazil, 2008-2016)

.

| Cancer Type | Algorithm | R2 in Overall Set | Number of Clusters Identified | Number of Clusters Identified  (p<0.05) |
| --- | --- | --- | --- | --- |
| Total Cancer | XGB | 0.66 | 3 | 3 |
| Total Cancer | Random Forest | 0.65 | 4 | 4 |
| Total Cancer | pSVM | 0.61 | 4 | 4 |
| Total Cancer | LASSO | 0.59 | 12 | 12 |
| C15 - Esophagus | XGB | 0.32 | 5 | 5 |
| C15 – Esophagus | Random Forest | 0.32 | 5 | 5 |
| C15 – Esophagus | pSVM | 0.30 | 8 | 8 |
| C15 – Esophagus | LASSO | 0.24 | 5 | 5 |
| C16 – Stomach | XGB | 0.13 | 1 | 1 |
| C16 – Stomach | Random Forest | 0.15 | 1 | 1 |
| C16 – Stomach | pSVM | 0.08 | 5 | 5 |
| C16 – Stomach | LASSO | 0.08 | 5 | 5 |
| C22 – Liver | XGB | 0.07 | 1 | 0 |
| C22 – Liver | Random Forest | 0.08 | 1 | 0 |
| C22 – Liver | pSVM | 0.07 | 1 | 0 |
| C22 – Liver | LASSO | 0.07 | 1 | 0 |
| C25 – Pancreatic | XGB | 0.25 | 1 | 0 |
| C25 – Pancreatic | Random Forest | 0.27 | 1 | 0 |
| C25 – Pancreatic | pSVM | 0.25 | 1 | 0 |
| C25 – Pancreatic | LASSO | 0.25 | 1 | 0 |
| C34 – Lung | XGB | 0.51 | 1 | 0 |
| C34 – Lung | Random Forest | 0.53 | 1 | 0 |
| C34 – Lung | pSVM | 0.45 | 5 | 5 |
| C34 – Lung | LASSO | 0.45 | 7 | 7 |
| C50 – Breast | XGB | 0.24 | 1 | 0 |
| C50 – Breast | Random Forest | 0.26 | 1 | 0 |
| C50 – Breast | pSVM | 0.24 | 1 | 0 |
| C50 – Breast | LASSO | 0.24 | 1 | 0 |
| C61 – Prostate | XGB | 0.11 | 1 | 0 |
| C61 – Prostate | Random Forest | 0.12 | 1 | 0 |
| C61 – Prostate | pSVM | 0.08 | 1 | 0 |
| C61 – Prostate | LASSO | 0.07 | 1 | 0 |
| C71 - Brain | XGB | 0.15 | 1 | 0 |
| C71 – Brain | Random Forest | 0.16 | 1 | 0 |
| C71 – Brain | pSVM | 0.15 | 1 | 0 |
| C71 - Brain | LASSO | 0.15 | 1 | 0 |
| C80 – Unsp. Location | XGB | 0.11 | 7 | 7 |
| C80 – Unsp. Location | Random Forest | 0.12 | 8 | 8 |
| C80 – Unsp. Location | pSVM | 0.09 | 10 | 10 |
| C80 – Unsp. Location | LASSO | 0.09 | 7 | 7 |
